# Repeat endoscopic intermuscular dissection of the visible scar after noncurative endoscopic intermuscular dissection of a rectal neuroendocrine tumor

**DOI:** 10.1055/a-2261-7919

**Published:** 2024-03-01

**Authors:** Elena De Cristofaro, Jérôme Rivory, Louis Jean Masgnaux, Timothée Wallenhorst, Jérémie Jacques, Pierre Lafeuille, Mathieu Pioche

**Affiliations:** 1 Gastroenterology Unit, Department of Systems Medicine, University of Rome Tor Vergata, Rome, Italy; 2 Gastroenterology and Endoscopy Unit, Edouard Herriot Hospital, Hospices Civils de Lyon, Lyon, France; 3 Gastroenterology and Endoscopy Unit, Pontchaillou University Hospital, Rennes, France; 4 Gastroenterology and Endoscopy Unit, Dupuytren University Hospital, Limoges, Limoges, France

Endoscopic resection with advanced techniques such as endoscopic submucosal dissection is the first-line treatment for small rectal neuroendocrine tumors (NETs) 1 . When initial resection is not curative (R0), systematic rectal revision seems relevant in order to remove the scar 2 . Recently, endoscopic intermuscular dissection (EID) has been described for increasing the deep resection margin and R0 rate 3 4 .

In this case we report the benefits of EID for removing the scar after a previous EID for rectal NET.

A 66-year-old patient was referred to our center for removal of a suspected rectal NET ( Video 1 ). An EID was indicated to optimize the deep resection margin. After circumferential incision and trimming, an incision of the circular muscular layer was performed, with longitudinal muscular layer exposure. We placed an adaptive traction device (A-TRACT 2; Hospices Civils de Lyon, France) to improve the exposure of the intermuscular plane. After cutting three-quarters of the lesion, proper traction was re-established by tightening the A-TRACT, and the resection was performed without damage to the longitudinal muscular layer ( Fig. 1 **a,b** ).

Repeat endoscopic intermuscular dissection after noncurative resection with traction strategies.Video 1

**Fig. 1:**
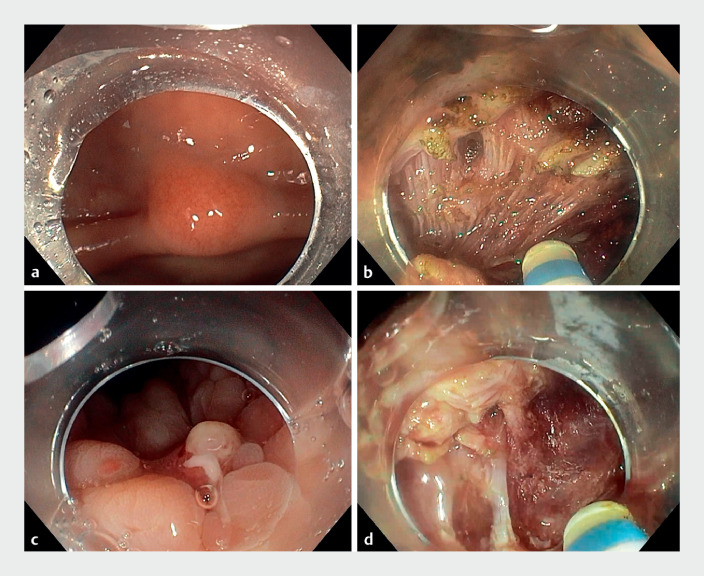
Endoscopy images for resection of a rectal neuroendocrine tumor (NET) using endoscopic intermuscular dissection. **a** The rectal NET. **b** Exposure of the intermuscular plane with the A-TRACT strategy. **c** The scar of the resected NET. **d** Exposure of the intermuscular plane with the double-clip traction strategy.

Histopathology showed a grade 1 well-differentiated NET, with deep free margins but minor contact with the lateral edge. For this reason, an endoscopic revision was indicated after 4 months, and a second EID was performed to remove the scar. A double-clip traction with clip on the circular layer was applied to improve the exposure of the intermuscular space ( Fig. 1 **c,d** ). The procedure was completed in 30 minutes, without adverse events. Histopathology did not reveal residual disease on the scar.

To our knowledge, this is the first described case in which EID was performed after a previous noncurative EID for a rectal NET. The use of traction strategies, especially of a dedicated adaptive traction device such as that used here, could facilitate the intervention, allowing better exposure of the intermuscular plane.

Endoscopy_UCTN_Code_TTT_1AO_2AC

## Notes

Endoscopy E-Videos https://eref.thieme.de/e-videos
*E-Videos* is an open access online section of the journal *Endoscopy* , reporting on interesting cases and new techniques in gastroenterological endoscopy. All papers include a high-quality video and are published with a Creative Commons CC-BY license. Endoscopy E-Videos qualify for HINARI discounts and waivers and eligibility is automatically checked during the submission process. We grant 100% waivers to articles whose corresponding authors are based in Group A countries and 50% waivers to those who are based in Group B countries as classified by Research4Life (see: https://www.research4life.org/access/eligibility/ ). This section has its own submission website at https://mc.manuscriptcentral.com/e-videos .
